# Emerging vaccine strategies against the incessant pneumococcal disease

**DOI:** 10.1038/s41541-023-00715-w

**Published:** 2023-08-17

**Authors:** Jeremy A. Duke, Fikri Y. Avci

**Affiliations:** 1 Sanofi, Suite 300, 2501 Discovery Drive, Orlando, FL 32826 USA; 2 Department of Biochemistry, Emory Vaccine Center, Emory University School of Medicine, Atlanta, GA 30322 USA

**Keywords:** Conjugate vaccines, Bacterial infection, Bacterial infection

## Abstract

The incidence of invasive pneumococcal disease (IPD) caused by infection with the pathogen *Streptococcus pneumoniae* (*Spn*) has been on a downward trend for decades due to worldwide vaccination programs. Despite the clinical successes observed, the Center for Disease Control (CDC) reports that the continued global burden of *S. pneumoniae* will be in the millions each year, with a case-fatality rate hovering around 5%. Thus, it is a top priority to continue developing new *Spn* vaccination strategies to harness immunological insight and increase the magnitude of protection provided. As emphasized by the World Health Organization (WHO), it is also crucial to broaden the implementation of vaccines that are already obtainable in the clinical setting. This review focuses on the immune mechanisms triggered by existing pneumococcal vaccines and provides an overview of the current and upcoming clinical strategies being employed. We highlight the associated challenges of serotype selectivity and using pneumococcal-derived proteins as alternative vaccine antigens.

## Introduction

Of the many bacterial pathogens responsible for widespread human illness, *Streptococcus pneumoniae* continues to be a significant threat, despite the introduction of vaccine programs to abrogate the spread of the disease four decades ago. Widespread asymptomatic colonization of the bacterium within the host nasopharynx gives rise to invasive disease as the body’s immune system has a reduced ability to act against the virulence factors of *Spn*, primarily the capsular polysaccharide (CPS) that coats the outer surface^1^. The distinct chemical structure of each CPS defines the approximately 100 serotypes known to comprise *Spn* as a species^2^. The heterogeneity in the monosaccharide building blocks and how they are linked and modified by the bacterium allows for specific antibody binding to each CPS, hence serotyping^2,3^. While regional differences exist in dominant serotype colonization and infection, 20 to 25 predominant serotypes are responsible for approximately 90% of IPD cases^4,5^.

IPD caused by *Spn* infection can take multiple clinical manifestations throughout the host, depending on the anatomical location bacterial pathogenesis occurs. Most commonly observed is lung infection leading to pneumonia, as the opportunistic bacteria spread through horizontal dissemination from the nasopharynx and colonize without immune clearance^6^. Continued propagation leads to more severe pathologies, with acute infection in the blood leading to septicemia or bacteremia and spinal cord and brain infections leading to meningitis^6,7^. These severe pathologies occur in nearly one-third of patients hospitalized with *Spn* infection, with survivors experiencing further sequelae, including the increased occurrence of neurological and cardiovascular defects and reduced lifespan^8^. Additionally, coinfection with IPD in hospitalized influenza or COVID patients significantly increases intensive care and fatalities^9–11^. A systematic analysis of lower respiratory infections found the global burden of *Spn* in 2016 to be an estimated 15 cases per 100,000 persons per year, or 1.1 million deaths resultant of pneumococcal infection^12^. Given the dire issue of IPD morbidity and mortality worldwide, preventing colonization through vaccination remains a high priority.

This review covers the current strategies used to develop pneumococcal vaccines and the findings related to their immune mechanisms. Additionally, we explore the clinical and manufacturing challenges associated with these vaccines. Finally, we highlight alternative protective antigens with the potential to provide broad protection in future clinical preparations.

### Immune mechanisms induced by pneumococcal vaccines

Phagocytosis is the primary mechanism for the immune clearance of pneumococcal colonization by the host^13,14^. CPSs help bacterial pathogens evade immune-mediated clearance by inhibiting complement activation and preventing phagocytosis^15,16^. On the other hand, their surface exposure and distinct chemical structures allow for the exploitation of CPSs in vaccine design as antigens to induce opsonophagocytic killing of bacteria and elicit protective immune responses against encapsulated bacterial pathogens^13,14^. Serotype-specific, antibody-mediated protection from pneumococcal disease was established as early as the beginning of the 20^th^ century, with modern vaccination programs seeing incredible success in the clinical space by seeking to eliminate the colonization of specific serotypes^17,18^. This goal has been pursued by building immunity against the CPS expressed on the surface of serotypes found to be most burdensome. The earliest iterations of this vaccine used purified, bacterial-derived CPS to activate the immune system, which is incapable of initiating the adaptive immune response and thus limited the vaccine’s effectiveness to eliciting predominantly short-living, low-affinity IgM antibody responses that are significantly reduced after six months in most serotypes, and back to baseline immunity within 24 months^19,20^.

The immunological mechanism harnessed using CPS alone as the antigen meant a specific clone of B cell capable of CPS recognition through its B cell receptor leads to the secretion of IgM antibodies^19^. However, most pure CPSs cannot trigger T cell help to induce antibody class switching, affinity maturation, and memory B and T cell production, all important for effective and prolonged protection^19,21–23^. An important milestone in vaccine design has been the introduction of conjugation technology in clinical vaccine programs^24,25^. The covalent conjugation of the CPS to an immunogenic carrier protein yielded what is now known as glycoconjugate vaccines^24,25^. Glycoconjugate vaccines induce an adaptive immune response targeting the CPS, IgM to IgG antibody class switching, affinity maturation, and immune memory, and are significantly more protective than pure polysaccharide vaccines^26,27^. While these vaccines have drastically reduced pneumococcal diseases globally, their variable immunogenicity, batch-to-batch inconsistency, and heterogeneous compositions manifested significant clinical shortcomings^24,25^. For example, numerous studies have shown that serotype 3 is highly unresponsive to the current pneumococcal multi-valent conjugate vaccine PCV13^28–31^, and incidence rates of serotype 3 continue to rise^32^. While *Spn*3 accounts for more than 10% (and increasing) of morbidity associated with IPD, it is responsible for approximately 25% of sepsis caused by *Spn*, and even more dramatically, IPD caused by serotype 3 has a 30% higher mortality rate than other serotypes^33–35^. Recent epidemiology work in Germany reveals serotype 3 as the most prevalent serotype identified in cases of community-acquired pneumonia, all of which required hospitalization^36^. Another epidemiology work in Portugal evaluated the impact of continued PCV13 vaccine use in pediatric IPD and reported serotype 3 as the most frequently detected serotype^37^. The need to modernize their design and development processes and generate structurally and functionally homogenous vaccines with increased efficacy offered opportunities to elucidate molecular and cellular mechanisms of how they induce adaptive humoral immune responses^24,25^.

A comprehensive elucidation of glycoconjugate vaccine-induced immune mechanisms was reported over a decade ago by ref. ^38^. According to this model, glycoconjugate vaccines stimulate strong adaptive immune responses through carbohydrate-specific T cells, or Tcarbs^38,39^. By covalently attaching CPS to an immunogenic protein, a shift in the immunological mechanism harnessed occurs, with the conjugate molecule being trafficked to the endolysosomes and degraded through the activity of reactive oxygen or nitrogen species as well as enzymatic hydrolysis, ultimately resulting in discrete glyco-epitopes that bind to the major histocompatibility complex protein, MHCII, and are presented by antigen-presenting cells for CD4 + T helper cell recognition^38–40^. This processing and presentation event is possible as the carrier protein contains sequences capable of association with MHCII, and the depolymerized CPS epitopes covalently bound to the peptide fragments on the MHCII groove enable the presentation of CPS to CD4 + T cells with a T cell receptor recognizing processed CPS as its epitope^38,40^. The immune synapse formed between the B and T cell facilitates the adaptive immune response through the differentiation of B cells to high-affinity IgG-producing plasma cells and memory cell formation^13^. The initial mechanistic study on glycoconjugate vaccines was followed by additional studies that strengthened and further elaborated the Tcarb-mediated immune responses induced by glycoconjugate vaccines^41–43^. In one study, glycoconjugates of serotype 3 *Streptococcus pneumoniae* CPS (Pn3P) were employed to provide evidence for the functional roles of Pn3P-specific CD4 + T cells that induce Pn3P-specific immunoglobulin responses in a Tcarb-dependent manner^40^. More recently, glycoconjugate vaccines composed of Vi antigen from *Salmonella typhi*, Group B *Streptococcus* type 1b, and *H. influenza* type b were shown to induce Tcarb-mediated adaptive humoral immune responses^41^. In the same study, conjugates of group C polysaccharide from *Neisseria meningitidis* failed to induce humoral immunity through Tcarbs due to complete depolymerization of the poly-sialic acid structure during antigen processing.

Elucidating the immune mechanisms to facilitate a protective immune response forms the knowledge basis for a forward-thinking approach to new-generation vaccine design. One example of knowledge-based vaccine design exploiting the antigen processing and presentation mechanisms elicited by glycoconjugate vaccines came from our studies, where we proposed the initial Tcarb model^38^. A prototype glycoconjugate vaccine designed to enrich for MHCII glyco-epitopes elicited up to two orders of magnitude higher IgG titers compared to a traditional glycoconjugate preparation and complete protection in a mouse challenge study.

### Correlates of protection

Vaccine efficacy and the degree to which protection is garnered against specific serotypes must have a normalized metric to quantify immunity and determine noninferiority between the different vaccines manufactured. These metrics are collectively known as correlates of protection and are used to measure the degree of humoral response and the functional capability of the antibodies produced after vaccination^44^. The simplest quantifiable method to evaluate vaccine efficacy is by determining the robustness in the humoral response generated at predetermined time points throughout the vaccination schedule. This is typically performed through an Enzyme-Linked Immunosorbent Assay, or ELISA, where the particular CPS antigen or strain of *Spn* is immobilized and incubated with vaccinated serum, allowing for the serotype-specific antibodies to bind. ELISA provides a broad readout for the concentration and classes of CPS-specific antibodies produced through vaccination. Opsonophagocytosis is the primary mechanism of immune clearance against *Spn* and the current gold standard metric of pneumococcal vaccine efficiency is the Opsonophagocytic Killing Assay (OPKA, sometimes also referred to as OPA), in which the efficiency of immune-mediated killing elicited by vaccination is gauged^14^. In this assay, the specific serotype of interest is co-cultured with phagocytic cells and immunized serum that will lead the immune cells to engulf and kill the bacteria. Using these correlates of protection is how vaccine noninferiority criteria are met, with preclinical and clinical studies being performed against current standards of care to measure if the humoral and functional response generated through vaccination is comparable in animal models and humans.

### Development of current clinical pneumococcal vaccines

One of the earliest iterations of an *Spn* vaccine, Pneumovax® PPSV23, came into the market in 1983 and was formulated as a multivalent vaccine using purified *Spn* CPSs isolated from the 23 serotypes responsible for the majority of IPD cases (Table 1)^45^. It was found to be efficient in adult and elderly populations, but poorly immunogenic in children and immunocompromised patients and had the drawback of requiring multiple doses as the efficacy of vaccine-elicited protection would wane without the ability to generate T cell-dependent immunity. The lack of protective immunity in the populations at highest risk of IPD and the variable responses and waning of immunity over time in the elderly represented an important challenge concerning vaccine-elicited protection against *Spn*^46,47^. In 2000, the multivalent Prevnar™ PCV7 was introduced to protect against the seven most virulent serotypes of *S. pneumoniae* through the preparation of individual serotype conjugates that are prepared and combined into multivalent formulations. Using purified CPSs that are first chemically modified to enable covalent attachment to CRM_197_, a genetically detoxified form of diphtheria toxin, the first commercially available conjugate vaccine against *Spn* was generated (Table 1). Updated versions of PCV7 have been released, covering additional serotypes from the 13-valent PCV13 up to the most recent 20-valent PCV20 (Table 1). Other carrier proteins have been explored, with the 10-valent Synflorix™ an example of a clinically available PCV that uses multiple carriers in its formulation, tetanus toxoid, and protein D from nontypeable *H. influenzae*.

**Table 1:** Clinical pneumococcal vaccines approved or in trial.

Vaccine	Manufacturer	Serotypes	Type	Clinical Status
PPSV23 (PNEUMOVAX®23)	Merck	1, 2, 3, 4, 5, 6B, 7F, 8, 9N, 9V, 10A, 11A, 12F, 14, 15B, 17F, 18C, 19A, 19F, 20, 22F, 23F, and 33F	CPS alone	Approved
PPV23	Sinovac Biotech Ltd.	1, 2, 3, 4, 5, 6B, 7F, 8, 9N, 9V, 10A, 11A, 12F, 14, 15B, 17F, 18C, 19A, 19F, 20, 22F, 23F, and 33F	CPS alone	Approved* *China Only
PCV10 SYNFLORIX	GSK	1, 4, 5, 6B, 7F, 9V, 14, 18C, 19F, and 23F	CPS-protein conjugate	Approved* *EU Only
PCV7 (PREVNAR 7™)	Pfizer	4, 6B, 9V, 14, 18C, 19F, and 23F	CPS-protein conjugate	Approved
PCV13 (PREVNAR 13™)	Pfizer	1, 3, 4, 5, 6A, 6B, 7F, 9V, 14, 18C, 19A, 19F, and 23F	CPS-protein conjugate	Approved
PCV20 (PREVNAR 20™)	Pfizer	1, 3, 4, 5, 6A, 6B, 7F, 8, 9V, 10A, 11A, 12F, 14, 15B, 18C, 19A, 19F, 22F, 23F, and 33F	CPS-protein conjugate	Approved
V114 (VAXNEUVANCE™)	Merck	1, 3, 4, 5, 6A, 6B, 7F, 9V, 14, 18C, 19A, 19F, 22F, 23F and 33F	CPS-protein conjugate	Approved
WEUPHORIA	WALVAX®	1, 3, 4, 5, 6A, 6B, 7F, 9V, 14, 18C, 19A, 19F, and 23F	CPS-protein conjugate	Approved
PNEUMOSIL	Serum Institute of India	1, 5, 6A, 6B, 7F, 9V, 14, 19A, 19F, and 23F	CPS-protein conjugate	Approved
V116^124^	Merck	3, 6A, 6C, 7F, 8, 9N, 10A, 11A, 12F, 15A, 15B, 15C, 16F, 17F, 19A, 20, 22F, 23A, 23B, 24F, 31, 33F, and 35B	CPS- protein conjugate	Phase 3
VAX-24^58^	Vaxcyte	1, 2, 3, 4, 5, 6A, 6B, 7F, 8, 9N, 9V, 10A, 11A, 12F, 14, 15B, 17F, 18C, 19A, 19F, 20B, 22F, 23F, and 33F	CPS-protein conjugate	Phase 3
AFX3772^60^	GSK + Affinivax	1, 3, 4, 5, 6A, 6B, 7F, 9V, 14, 18C, 19A, 19F, 23F, 2, 8, 9N, 10A, 11A, 12F, 15B, 17F, 20B, 22F, and 33F	MAPS™	Phase 3
CPS-EPA^iGTcc^ ^63^	Omniose	8, 9V, and 14	Bioconjugate	Preclinical

In preparing these PCVs, there is a need for the chemical modification of CPS to an activated state prior to covalent attachment to the protein carrier due to the lack of reactive chemical handles on nearly all CPS used by *Spn*. Most conjugate formulations are developed by the initial oxidation of cyclic diol structures found within the CPS, using sodium periodate to modify the CPS and form reactive aldehyde groups capable of undergoing reductive amination with the surface-exposed lysine residues on a protein carrier to form a permanent bond^48^. This empirical method of conjugate formation and the large size of bacterially derived CPS leads to large globular structures as a lattice of CPS and carrier protein is formed through chemical attachment, limiting the degree of analytical approaches that can be used to define and characterize conjugates and evaluate clinical readiness^49,50^. In this regard, conjugates are analyzed for non-conjugated protein and polysaccharide content, and a ratio of protein to polysaccharide is determined to establish batch-to-batch consistency. Manufactured vaccines may have significant variability in physiochemical properties due to the different carrier proteins and the conjugation chemistry employed by each manufacturer. Additionally, the structural heterogeneity of conjugate vaccine products may arise among different batches of the same vaccine due to the inherent complexity of chemical conjugation strategies.

### Recent developments in pneumococcal vaccine technology

In most iterations of current pneumococcal conjugate vaccines used in the clinic, an empirical approach is used to generate the molecules by activating the CPS for its covalent attachment to carrier proteins^48^. While this synthesis route works, it has shortcomings, such as overlooking the tentatively deleterious role the chemical activation of CPS can play, and that non-specific conjugation to the carrier can lead to a product that does not enable MHC presentation of key antigens^51,52^. The desire to combat the challenges with the current conjugation methodologies and enable better physiochemical characterization have prompted companies and academic research groups to explore the development of rationally defined and designed conjugate molecules. In the current clinical space, advances toward combating these shortcomings are being made with new formulations and phase trials to enhance conjugate vaccines' immune potential and structural homogeneity (Table 1).

Pneumosil is a recently developed and approved 10-valent conjugate vaccine comparable to other marketed PCVs in efficacy that uses an activation method that is not dependent on periodate oxidation, thereby potentially reducing the possible damage to CPS antigens that oxidation may have^53^. Conjugate preparations are made using CPS conjugated through 1-cyano-4-dimethylaminopyridinium tetrafluoroborate (CDAP) chemistry to CRM_197,_ an approach that does not permanently alter the chemical structure of CPS outside of the points of conjugation^54,55^. This has the benefit of not significantly altering important physiochemical characteristic of the CPS.

Another strategy in development, VAX-24, tackles 24 serotypes through the preparation of conjugates using click chemistry functionalized CPS and the proprietary carrier eCRM, a CRM_197_ that has been modified to be expressed with replacement of lysine residues along the surface-exposed T cell epitopes into a non-canonical amino acid capable of specific reaction with the derivatized CPS^56^. This approach allows for the inclusion of site-selective conjugation that does not interfere with protein folding, thus keeping key tertiary structure features while enriching for CPS conjugate epitope sites^57^. Pre-clinical trials comparing VAX-24 to PCV13 and PPSV23, the standards of care at the time, illustrated comparable levels of humoral immune response and robust correlates of protection in animal models^58^. VAX-24 recently finished Phase 2 trials and has been designated as Breakthrough Therapy by US Food and Drug Administration (FDA) going into Phase 3, thus representing the value of analytically defined conjugate molecules. Critical T cell epitopes are claimed to be left undisturbed by the ability to specifically conjugate the CPS to the carrier at key points while minimizing conjugate heterogeneity and maximizing protein to polysaccharide content, all previously noted considerations that need to be addressed in the next generation of conjugate vaccines^25^.

AFX3772 is another versatile vaccine formulation that uses novel CPS and carrier modification to elicit protection against IPD in 24 serotypes, including *Spn* protein antigens^59^. The foundation of this approach is to use the Multiple Antigen-Presenting System (MAPS™) and generate macromolecular complexes consisting of biotin-modified CPS noncovalently attached to rhizavidin-fused carrier proteins. The strong interaction between biotin and rhizavidin functionally fuses the two components akin to a traditional conjugate but with a higher degree of control and versatility towards what is included in the complex^59^. In a combined Phase 1/2 study to evaluate safety and efficacy in healthy adults, AFX3772 exhibited a more robust antibody response than those generated upon vaccination with PCV13 and PPSV23 while maintaining a similar safety profile^60^. Due to the positive findings from this combined trial, the FDA granted AFX3772 the designation of Breakthrough Therapy as a preventative against IPD to adults aged 50 and older.

The vaccine candidates described thus far require a laborious process of individual isolation of the CPS antigen from bacteria, expression of the carrier structure to which it will be attached, subsequent modification and conjugation, and purification of a finalized product. To streamline the conjugation process and significantly decrease the cost of generating conjugate molecules, an alternative strategy is being explored in the preclinical space called Protein Glycan Coupling Technology, or bioconjugation. This approach clones the bacterial machinery for expressing the CPS, the carrier, and the enzyme that covalently attaches the two into *E. coli* to generate vaccine molecules, which are then capable of being purified and used directly, a strategy that has been used against other pathogenic bacteria previously^61^. In the context of *Spn*, it incorporates an oligosaccharyltransferase termed PglS that enables the covalent attachment of glucose-reducing end glycan structures, the most commonly found amongst CPS serotypes, onto a carrier protein that has a site for attachment by PglS^62^. This approach has the added benefit of a more homogenous conjugate identity, with the CPS attaching at the specific epitope sites PglS uses and through a singular attachment point on each polysaccharide’s reducing end. Evidence for the efficacy has been demonstrated through the generation of trivalent formulations that can generate a humoral immune response equal to PCV13, with a further study thoroughly characterizing the protection from sepsis and prevention of colonization of serotype 8^63,64^.

### Serotype selection in clinical formulations

Determination of the specific serotypes selected for inclusion in commercial formulations is an essential consideration in combating IPD, as there is some known heterogeneity in the reported prevalence and distribution of the serotypes on a global scale. Insight into the incidence rates caused by specific strains is critical in manufacturing vaccines, as this will drive vaccine policy and protective coverage. Overall, seven serotypes (1, 5, 6A, 6B, 14, 19F, and 23F) have been found to be the most common IPD-causing strains, with global analyses illustrating that two-thirds of all cases are isolates of these strains^65^. By further increasing the number of serotypes covered in PCV formulations, most strains responsible for IPD can be targeted. Prevnar 13®, the 13-valent PCV, was able to cover 76% of strains responsible for children under the age of five, with an estimated 78% reduction in IPD vaccine-related serotypes after introduction in the United States^65^. The updated 20-valent iteration now covers approximately 90% of total IPD serotypes^66^. The regional burden of particular serotypes varies, with current formulations more specifically protecting North American and European populations, whereas Asian and African IPD cases are due to a wider variety of serotypes^65^. Considerations must be made regarding what serotypes are selected for, as the current cumulative approach of continually adding serotypes in formulation does not account for possible evolutionary dynamics or the possible dampening of the immune response as the breadth of antigens introduced each vaccine increases. Further limitations exist outside the approach used to generate conjugate molecules and the selection of serotypes in formulations that drive vaccine policy^12,67^.

Serotype replacement is a phenomenon known to occur in response to vaccination as the current formulations are all dependent on targeting combinations of serotypes, with little to no meaningful amount of cross-protection elicited towards those not included^68,69^. While incidence rates and geographical trends play a major role in selecting serotypes to be included in the vaccine preparations, it results in a shift of *Spn* population dynamics as non-vaccine serotypes flourish and evolve outside of the natural fluctuations that are expected^70,71^. Most observations in this regard come from trends after the first-generation pneumococcal conjugate vaccines that targeted a smaller number of serotypes, as the later 13-, 15-, and 20-valent formulations have only more recently been made available. In one such study following a cohort of 383 children that had received the 7-valent conjugate vaccine, the overall carriage of *Spn* within the nasopharynx did not dramatically alter over time, but rather an increase in the non-vaccine serotypes compensated for the dramatic decrease in the serotypes vaccinated against^72^. Further, in a comprehensive analysis of pneumococcal isolates spanning pre- and post-PCV7 introduction by the CDC, it was found that a 1.5-fold increase of non-vaccine serotypes occurred over three years^73^. In the limited data looking at overall pneumococcal incidence before and after the introduction of the 7- and 13-valent conjugate vaccine, it was found that a small number of non-included serotypes rapidly increased to causing 40% of IPD occurrence, despite a relatively small degree of colonization previously^74^. While these trends do not dramatically impact the morbidity of IPD, it is critical to consider serotype selection and fluctuation in clinical preparations moving forward.

An additional topic of concern relative to serotype selection is the occurrence of capsular switching due to variable genotypic information within a serotype and the ability to share DNA between strains, resulting in differential expression of the CPS^75^. The ability of *Spn* to carry differing genetic information that resulted in distinct CPS was first described decades ago before it was fully understood what a genetic cassette was^76^. In most cases of CPS expression, a specific gene locus containing multiple genes related to capsule production occurs, with the diversity found across *Spn* as a species a consequence of mutation over time and intraspecies recombination^77^. Introducing a selective pressure in the form of serotype-specific vaccination can result in changes in CPS induction to enable the survival of the *Spn* and continual colonization, and has been observed in multiple studies, albeit in small numbers^70,78–80^. While capsular switching does not significantly threaten overall vaccine efficacy, it can exacerbate the rise of non-vaccine serotypes and represents a larger risk in antibiotic gene transfer amongst *Spn* serotypes^81^.

### Alternative pneumococcal antigens

Despite the successes witnessed in the clinical space using CPS-containing vaccines, the serotype-directed approach has a major shortcoming: it can only protect against the individual serotypes accounted for in formulations. The outstanding serotype coverage obstacle is further compounded by the recent rise of non-encapsulated *Spn* strains that can colonize, albeit with no direct correlation to increased IPD cases in most circumstances^82^. With these considerations in mind, employing vaccine candidates based on conserved *Spn* surface proteins as the driving immunogen is an important area of focus that would expand the breadth of strain coverage while limiting the occurrence of capsular switching events. Towards this goal, multiple *Spn* surface proteins that could be harnessed as vaccine targets have been explored and thoroughly reviewed elsewhere^83^. Representative conserved vaccine candidates employed in clinical trials are summarized in Table 2 and below^83,84^.

**Table 2:** Representative protein-based vaccine candidates and trials (adopted from)^83^.

Protein	ClinicalTrials.gov Identifier	Phase	Status	Representative References
PhtD	NCT01767402	Phase I	Completed	^125^
PhtD-dPLY	NCT00896064	Phase II	Completed	-
PlyD1	NCT01444352	Phase I	Completed	^92^
PhtD	NCT01444001	Phase I	Completed	^102^
PcpA (Pneumococcal choline-binding protein A), PcpA + PhtD	NCT01444339	Phase I	Completed	^126^
PhtD + PcpA + PlyD1	NCT01764126 NCT01446926	Phase I	Completed	^103^
dPLY + PsaA (Pneumococcal surface adhesin A) + 24 CPS	NCT03803202	Phase I/II	Completed	-
	NCT04525599	Phase I	Active	-
PspA + PlyD	NCT04087460	Phase Ia	Active	-
dPLY + PhtD + PCV10	NCT01262872	Phase II	Completed	^93–95^

The longest studied of pneumococcal protein immunogens is the cholesterol-dependent cytolysin, pneumolysin (PLY)^85^. PLY has been observed in nearly every IPD-causing strain, emphasizing the value such a vaccine target could bring to the clinical space^86^. The full-length PLY protein was found to provide protection against multiple *Spn* serotypes but exhibited cytotoxic behavior, leading to the exploration of detoxified mutants^87,88^. Genetically and chemically detoxified PLY derivatives (i.e., dPLY and PlyD), along with the immunogenic domains of the protein, are explored as vaccine targets, with positive results observed against several strains in early pre-clinical trials and clinical trials (Table 2)^89–95^.

Choline-binding proteins are major bacterial virulence factors, pneumococcal surface protein A (PspA) being among the most studied protein vaccine candidates as nearly all the clinically isolated *Spn* strains have been found to express it^96,97^. However, its large heterogeneity observed in domain sequences results in variability between clinical isolates^98^. Using conserved sequences amongst closely related domain clades and multivalent protein formulations, protective efficacy has been shown in early animal models and clinical trials to further drive PspA as a strong candidate for an alternative multi-serotype immunogen^99,100^.

Another major avenue of interest is the pneumococcal histidine triad protein D (PhtD) surface protein. First isolated and tested more than two decades ago, it was found to protect against sepsis in three highly virulent strains of *Spn*^101^. A strong push to use PhtD in the clinic led to phase trials both as a standalone immunogen and in concert with other protein targets, with results indicating the strong possibility of use in a protective vaccine^102–104^.

Using multiple protein antigens the most success has been found in the clinic, with successful early-stage clinical trials showing protection against specific serotypes of *Spn*^83^. These results, together with the expectedly lower cost of production, make *Spn* antigens an appealing vaccine candidate, especially when multiple are used together, albeit the issue of serotype non-inferiority against standard conjugate vaccines must be overcome. Expanding on the goal of developing a conserved/universal pneumococcal protein vaccine has led to the adaptation of a reverse vaccinology approach, wherein bioinformatics tools are applied to the *Streptococcus pneumoniae* genome to identify novel and tentatively immunogenic proteins^105,106^. These studies have shown promise in the discovery of new and promiscuous protein antigens capable of antibody generation specific to *Spn* but will need to be further refined and scrutinized given a lack of protection in murine sepsis challenge in vivo. The envisioned potential of this approach could work in concert with other important avenues of vaccinology research, including the emergent use of novel delivery systems that broaden the administration routes available and can act as a self-adjuvant^107,108^, which has been explored in one pre-clinical study using PspA^100^.

## Discussion

The introduction of pneumococcal conjugate vaccine programs has profoundly impacted mortality and disease severity of IPD, with the need to push these programs further and address the limitations that have begun to arise. One remaining challenge lies in the methodology in which conjugate preparations are generated, in most cases working in an empirical approach to synthesize PCV products without taking insight from the immunological mechanisms at work. In these vaccines, there is an element of randomness in terms of where on the carrier protein and CPS conjugation is going to occur, with the issue further compounded by the as-of-yet unknown effect that polysaccharide size, chemical identity, and post-conjugation conformation play on immune responses^25,41^. The current conjugation methods add an additional degree of uncertainty, as there is no systematic assessment looking at all the different chemical approaches amongst the different serotypes and the relevance that linker molecules or degree of attachment can play in getting the CPS to be recognized by the B and T cells.

The selection of carrier protein employed plays a key role, as it enables the shift to an adaptive immune response, but with multiple carriers being used in the clinic, it isn’t clear if there is an ideal choice or how much the carrier itself is going to contribute to the protective action of the vaccines. Understanding carrier protein immune responses and thus enabling the use of carrier-derived peptide epitopes in developing novel knowledge-based vaccines may help combat persisting problems in glycoconjugate vaccine design. A potential concern is carrier-induced epitope suppression (CIES)^109–111^, wherein antibody response to the polysaccharide portion of the glycoconjugate vaccine can be inhibited due to pre-existing immune response to the carrier protein from prior immunizations. We postulate that reconstructing carrier proteins to form endolysosome-cleavable strings of polypeptides and their conjugation with polysaccharides is a novel approach to tackling CIES and the structural and functional heterogeneity of current conjugate vaccines. Toward this goal, we isolated and characterized new human MHCII-binging peptide epitopes derived from the two important vaccine immunogens TT and CRM197^112^. Harnessing carrier-derived peptides to generate multivalent carriers may yield knowledge-based, structurally, and functionally well-defined conjugate vaccines with robust, highly controlled, cost-effective production processes.

As an alternative or complementary strategy to conjugate vaccines, pneumococcal protein vaccines are being pursued in preclinical and clinical settings. These vaccines are designed to overcome the challenges associated with serotype-dependent PCVs and are postulated to work in conjunction with the conjugate vaccines currently employed in clinical practice. A serotype-independent protein vaccine has various advantages. It can be a cheaper option for children in developing countries, cover a wider range of geographical serotypes that cause IPD, and provide a new or alternative way to prevent recurrent infections. In the realm of manufacturing, it is imperative to note that protein-based antigens do not encounter the same hindrances associated with structural differences and variations between batches that are commonly observed in conjugate vaccines. Analytical and structural characterization tools such as mass spectrometry, NMR, and X-ray crystallography would allow for better characterization of protein vaccine products.

On the other hand, protein immunogens have several significant potential limitations as vaccine targets. First, they may or may not be present in most/all clinical isolates. Even if expressed in most clinical isolates, they may have significant sequence heterogeneity, rendering them poorly conserved. Another potential limitation of protein immunogens is their accessibility to opsonic antibodies on the bacterial surface. OPA is a major antibody-mediated immune response against extracellular bacterial pathogens, which makes CPSs an ideal vaccine target due to their accessibility to antibody binding. Along the same lines, protein immunogens mostly contain non-repeating conformational epitopes for antibody binding, making it challenging to raise protective antibody-mediated immunity, compared to CPSs, which possess repeating, conformationally less-restricted antigenic determinants. These limitations of protein immunogens may partially explain the difficulty in developing protein-based vaccines against extracellular bacterial pathogens. Regarding successful protein-based bacterial vaccine targets, non-toxic diphtheria and tetanus toxin variants effectively target the secreted toxins and inherently overcome the obstacles many protein vaccine targets face.

As a plausible conserved vaccine target, glycoproteins can offer the benefits of both polysaccharides and proteins by combining their antigenic and immunogenic properties. In *S. pneumoniae*, the Pneumococcal Serine-Rich Repeat Protein (PsrP) was established as a major virulence factor through large-scale mutagenesis screenings for lung infection^113^ and subsequently studied as a pathogenicity island^114^. PsrP is a very large, highly glycosylated, mucin-like adhesin that expands beyond the capsule^115^. Recently, multiple glycosyltransferases (GTs) responsible for PsrP glycosylation were shown to significantly impact PsrP’s biofilm formation and adhesion properties and lung infection in mice by intratracheal challenge^115^. Most recently, we investigated the prevalence and homology of PsrP globally, using the genomes of 13,454 clinically isolated pneumococci from the Global Pneumococcal Sequencing Project^116^. We found PsrP to be present in at least 50% of all strains, ranging up to over 70%, and it is heavily expressed in both vaccine and non-vaccine serotypes. Thus, formulating a new PsrP glycoprotein vaccine may serve the goal of a specific immune response that can provide broad protection against IPD globally. New and innovative conserved vaccine designs are being developed through preclinical research and clinical trials. These advancements, from animal testing to human testing, show potential for developing next-generation vaccines.

One area that requires particular attention is the introduction of new in vivo or ex vivo models for evaluating glycoconjugate vaccines. Current animal models with surrogate markers of protection (i.e., antibody titers and opsonophagocytic activity) can be inadequate and unreliably predictive of the responses of the target diverse human populations. Employing in vivo humanized mouse models to identify key helper T cell populations essential for vaccine efficacy may be highly important and innovative. Thankfully, the field of humanized mouse models is very rapidly progressing^117^. Alternatively, identifying human helper T cell populations essential for conjugate vaccine efficacy as a new immune correlate of protection may help tackle the obstacles associated with the limited current correlates of protection. To this end, understanding the molecular and cellular immune mechanisms induced by conjugate vaccines may yield a new immune correlate of protection to assess clinical vaccine products for their protective efficacy.

Overall, the brisk pace of development for pneumococcal vaccines has been remarkable, with numerous academic and industrial groups further elevating our understanding of how to control IPD worldwide. For many years, glycoconjugate vaccine formation was an unsystematic process of attaching immune-eliciting protein to the carbohydrate one wished to generate a response against. The current industry goals with novel vaccine strategies are primarily to develop *distinct* conjugation platforms with *better-controlled chemistry* and demonstrate *non-inferiority*, and not necessarily to garner new knowledge or improve on the efficacy of current conjugate vaccines. Understanding how conjugate vaccines work (i.e., what makes them antigenic and immunogenic) and why the current vaccines fail are critical parameters to achieve truly protective, widely applicable, and accessible vaccines against bacterial pathogens. By understanding the mechanism and process glycoconjugate vaccines undergo to elicit an immune response, a forward-thinking approach to future vaccines can begin. The goal is to develop a vaccine platform that is applicable to many microbial glycans because it is based on the basic immunologic mechanisms responsible for glycoconjugate processing, presentation, and T-helper cell reactivity. Our contribution towards this goal has been to establish new chemical and enzymatic conjugation strategies to develop chemoselective and structurally well-defined conjugative vaccines^51,118,119^; identify and characterize new human CD4 + T cell epitopes from vaccine immunogens^112^; develop a new structural model for the ligand binding of CPS specific protective antibodies^120^, and develop new chemical and enzymatic strategies to modulate CPSs for their optimum conjugation with carrier immunogens^121–123^. As the greater understanding of what drives immunological efficacy in PCVs grows, these insights can be further applied in the continued innovation in knowledge-based vaccine design.
